# Arthroscopic Assessment of the Incidence of Biceps Pulley Lesions Associated With Rotator Cuff Tears: An Observational Study

**DOI:** 10.7759/cureus.66593

**Published:** 2024-08-10

**Authors:** Mahmoud Kamal Eldin, Ehab Alieldin, Ahmed Ashour, Ahmed Ismail, Ahmed T Ashour, Saeed Shekedaf

**Affiliations:** 1 Trauma and Orthopedic, Alexandria Main University Hospital, Alexandria, EGY; 2 Trauma and Orthopedic, Barts Health Trust, London, GBR; 3 Trauma and Orthopedic, Queen Elizabeth Hospital Birmingham, Birmingham, GBR; 4 Trauma and Orthopedic, Bradford Royal Infirmary, London, GBR; 5 Trauma and Orthopedic, Elhadara University Hospital, Alexandria, EGY

**Keywords:** biceps tendon, observational studies, rotator cuff tears, biceps pulley lesions, arthroscopy

## Abstract

The shoulder joint houses a stabilizing structure called the biceps pulley. Biceps pulley lesions can trigger anterior shoulder pain and frequently coincide with rotator cuff tears, whose prevalence rises with age. In our study, we aim to assess the incidence of biceps pulley lesions associated with rotator cuff tears in patients undergoing arthroscopic repair, the possible associated factors, and whether MRI findings were correlated with them. This study was a prospective observational one conducted at Al-Hadra University Hospital. The patients aged 40 to 65 years were indicated for arthroscopic repair of a rotator cuff tear. We used IBM Corp. Released 2011. IBM SPSS Statistics for Windows, Version 20.0. Armonk, NY: IBM Corp. to conduct the analysis. A total of 60 patients were enrolled in the study. The mean age was 50.97 ± 6.90. The overall incidence of biceps pulley lesions was 85%. Older age was found to be significantly associated with increased incidence. On the other hand, gender, and the mode of injury (cuff tear) had no significant associations with the incidence. Also, formal MR had no significance in diagnosing biceps pulley lesions. The overall incidence of biceps pulley lesions in the current study was 85%. The older the patient with a cuff tear, the greater the incidence of finding a pulley lesion arthroscopically. Moreover, MRI did not have a significant role in diagnosing the biceps pulley lesions.

## Introduction

The biceps pulley is a capsuloligamentous complex, mainly composed of the coracohumeral ligament (CHL), the superior glenohumeral ligament (SGHL), supraspinatus (SSP) fibers, and subscapularis (SSC) tendons. The biceps pulley stabilizes the long head of the biceps tendon (LHBT) at the humerus bone's anterior aspect [1-5]. The lateral and medial pulleys can be visualized during the arthroscopy from the posterior portal, provided the arm is in 30° flexion and neutrally rotated. A posterolateral pulley injury is suggested by lateral translation of the LHB with the arm internally rotated as opposed to medial displacement with external rotation [6].

Biceps pulley lesions can result from acute trauma, repetitive microtrauma, and degeneration of the involved soft tissues or are associated with rotator cuff tears [7,8] or with internal anterosuperior impingement [7]. Such lesions are a common cause of pain in the anterior shoulder [9,10]. Arthroscopic examination of the injured biceps pulley reveals four types of morphologic differences at the site of insertion into the medial attachment point of the intertubercular groove, as described by Choi et al. [11].

Rotator cuff injury can refer to tendinopathy, partial tears, or even complete tears [12]. Every decade, there has been an increase in the severity and prevalence of rotator cuff tears, with up to 50% of people over 80 years old having rotator cuff tears [13]. In rotator cuff injuries, the biceps pulley may be strained by the unstable biceps tendon, which may compromise the SSC tendon's stability. It is essential to differentiate between normal variants and biceps pulley lesions affecting the anterosuperior injuries in rotator cuff tear cases [9].

Habermeyer et al. classification [7] are currently widely used for describing these lesions [10]. This classification consists of four different patterns. Pattern I refers to isolated lesions of the SGHL. Pattern II is an SGHL injury associated with a partial articular-sided SSP tendon tear. Meanwhile, pattern III involves SGHL injury associated with a deep surface tear in the SSC tendon, and pattern IV defines SGHL injury associated with partial articular-sided tears in both tendons of the SSC and SSP.

This study targeted the assessment of the incidence of biceps pulley lesions associated with rotator cuff tears in patients undergoing arthroscopic repair and the possible factors associated with such an incidence. Also, to evaluate the MRI findings correlated with them and their role in the diagnosis.

## Materials and methods

Study design and patient

This observational study was conducted at Al-Hadra University Hospital, Alexandria, Egypt, from October 2020 to December 2021, involving patients undergoing arthroscopic repair of rotator cuff tears. The study adhered to the STROBE statement for reporting observational studies. All patients provided informed consent to participate. Patients aged 40 to 65 years who were indicated for arthroscopic repair of rotator cuff tears were included. Patients who underwent open repair for rotator cuff tears had advanced arthritic changes of the glenohumeral joint or had other shoulder pathologies such as instability or calcifying tendonitis was excluded. All patients underwent thorough clinical, radiological, and arthroscopic evaluations. An MRI examination was mandatory for all cases to assess the pathology of the rotator cuff tear and to identify any biceps pulley lesions correlated with injuries observed during arthroscopic evaluation. The study followed the STROBE statement in reporting observational studies [14].

Surgical procedure

Surgery was performed with the patient in a beach chair position. Before arthroscopy, a standardized examination under general anesthesia evaluated the passive range of motion and glenohumeral instability. A 30° arthroscope was used in all cases. Diagnostic views of the joint were obtained through standard posterior and anterior portals. The long head of the biceps (LHB) tendon was visualized at the bicipital groove level through a flexed elbow and an elevated shoulder, using a probe to pull out the LHB. Internal rotation allowed evaluation of the medial pulley system and the adjacent subscapularis tendon. After intra-articular viewing, the scope was switched to the subacromial space, and a bursoscopy was performed to assess subacromial pathologies and evaluate the presence and size of rotator cuff tears. This was done with the scope through an anterolateral portal.

Classification and intervention

Habermeyer's classification was used to assess biceps pulley lesions. Factors determining the type of surgical intervention (tenotomy or tenodesis) included patient age, size of the rotator cuff tear, type of biceps pulley lesions, associated LHB tendon pathology, and surgeon experience. Tenodesis was primarily performed in patients with large or massive rotator cuff tears, while tenotomy was applied to older patients and those with concomitant LHB tendon affection.

Statistical analysis

Data analysis was conducted using IBM Corp. Released 2011. IBM SPSS Statistics for Windows, Version 20.0. Armonk, NY: IBM Corp. The Kolmogorov-Smirnov test verified the normality of the distribution. Qualitative data were described as numbers and percentages, while quantitative data were expressed as range (minimum and maximum), mean and standard deviation, or median and interquartile range (IQR). The significance level was set at 5%. Statistical tests used included the Chi-square test, Fisher's exact test, student's t-test, and Monte Carlo correction.

## Results

Demographic characteristics

The study included 60 patients who underwent arthroscopic repair of rotator cuff tears. The mean age was 50.97 ± 6.90, ranging from 40 to 65 years. There were 43 females and 17 males. The study included 47 patients with right-side rotator cuff tears and 13 patients with left-side cuff tears. Only one-third of the patients (20 patients) had associated medical conditions. A total of 35 cases (58.3%) had a degenerative tear of the rotator cuff, while 25 (41.7%) had a traumatic tear. As regards the morphology of the pulley on arthroscopic examination, 92.2% of cases with injured pulleys (47 of 51) showed type III by its subtypes (A, B, C, and D). In comparison, 7.8% of cases with an injured pulley (4 of 51) showed type IV. All details are shown in Table 1.

**Table 1:** Demographic characteristics of the included patients Quantitative data are presented as mean ± standard deviation, while qualitative data are presented as a number (percentage)

Characteristic	Data
Age	50.97 ± 6.90
Gender (Females)	43 (71.7%)
Side affected (Right side)	47 (78.3%)
Medical Comorbidities	
Diabetes Mellitus	20 (33.3%)
Hypertension	15 (25.0%)
Bronchial Asthma	10 (16.7%)
Degenerative tear of rotator cuff	35 (58.3%)
Arthroscopic morphological changes of pulley	
Type III	47 (92.2%)
Type IV	4 (7.8%)

Incidence of biceps pulley lesion

The overall incidence of biceps pulley lesions was 85%. According to Habermayer classification, 45 cases with injured pulleys (88.2%) were classified as Grade 2, one case (2%) as Grade 3, and five cases (9.8%) were classified as Grade 4.

Association between several factors with the incidence of an injured pulley and assessment of MRI findings in the diagnosis

Age was found to have statistical significance in the incidence of pulley lesions (p=0.002). The older the patient, the higher the incidence of an injured pulley. Meanwhile, there were no statistically significant associations between gender or the mode of injury (cuff tear) and the incidence. All cases in our study were subjected to formal MR for diagnosis of rotator cuff tear. None of these cases were reported to have any specific signs of biceps pulley lesions. Therefore, formal MR had no significance in diagnosing biceps pulley lesions. All details are shown in Table 2. 

**Table 2:** Table showing the association between several factors with the incidence of an injured pulley ': Independent Samples t-Test, ": Chi-Square Test, *: Statistically significant

Factors (N = 60)	Morphology of pulley		Tests used	p-value
	Intact (n = 9)	Injured (n = 51)		
Age (years)			-	-
Min. – Max.	40.0 – 65.0	41.0 – 65.0	3.695'	0.002*
Mean ± SD	50.97 ± 6.90	45.67 ± 4.15	3.695	0.002*
Median (IQR)	50.0 (45.5 – 56.0)	45 (40.0 – 65.0)	3.695	0.002*
Gender				
Males N (%)	3 (33.3%)	14 (27.5%)	0.130"	0.704
Females N (%)	6 (66.7%)	37 (72.5%)	0.13	0.704
Mode of Injury				
Degenerative N (%)	6 (66.7%)	29 (56.9%)	0.303"	0.722
Traumatic N (%)	3 (33.3%)	22 (43.1%)	0.303	0.722

Surgical management and different associations

A total of 43 cases with injured pulley (84.3%) were subjected to surgical intervention by tenotomy or tenodesis, while eight cases with injured pulley (15.7%) were not subjected to surgical management. Surgical intervention (tenotomy and tenodesis) was done in 23 cases of a degenerative cuff tear, while the biceps tendon was left without intervention in six cases. The surgical intervention was done in 20 cases of traumatic cuff tears, while the tendon was left intact in two cases. The mode of trauma did not have a significant association with the surgical management of the biceps tendon.

Eight cases were not subjected to any surgical intervention, while 15 cases underwent tenotomies and tenodesis at the age of 40-50 years. On the other hand, all cases aged 50 to 65 years old were subjected to surgical intervention either by tenotomy or tenodesis (Table 3).

**Table 3:** Table showing the association between surgical management options, age, and mode of injury X^2^ is a Chi-Square test

Factors (N = 51)	No Surgical Intervention (n=8)	Tenotomy (n=33)	Tenodesis (n=10)	X²	p-value
Age (years)					
40 – <50 N (%): 23 (45.1%)	8 (100%)	10 (30.3%)	5 (50%)	13.25	0.001
50 – 60 N (%): 19 (37.3%)	0 (0%)	15 (45.5%)	4 (40%)	6.094	0.04
>60 – 65 N (%): 9 (17.6%)	0 (0%)	8 (24.2%)	1 (10%)	2.409	0.419
Mode of Injury					
Degenerative N (%): 29 (56.9%)	6 (75%)	17 (51.5%)	6 (60%)	1.437	0.552
Traumatic N (%): 22 (43.1%)	2 (25%)	16 (48.5%)	4 (40%)	1.437	0.552

## Discussion

The current study found an overall incidence of biceps pulley lesions in 85% of cases. These results were comparable to those of Hawi et al. [15], who conducted a retrospective study in 2017 on 382 patients with rotator cuff tears and reported that 90.3% had an injured pulley system. Our study found the traumatic etiology of cuff tears in 43%, comparable to previous research reporting 44.6% [15]. In the current study, most of the cases with injured pulleys (88%) were classified as grade 2 according to Habermayer classification [7], which was different from Hawi et al. [15], which reported that most of the cases were classified as grade 4 and grade 2 with 43.8% and 36.5%, respectively.

In a prospective study [9] that analyzed the pulley in cases who underwent arthroscopic shoulder surgery for different causes to assess the prevalence of biceps pulley lesions properly, overall, they revealed a 32% prevalence of torn biceps pulleys and a 79% (53 of 67) of pulley tears in patients with rotator cuff tears. In their study [9], the overall incidence of biceps pulley lesions in cases with rotator cuff tears was 50.4% (53 of 105 patients), which is less than the incidence in the current study. This difference could be justified, as the current study investigated the incidence of biceps pulley lesions in only rotator cuff tears.

In a retrospective study [11] of 589 cases with rotator cuff tears demonstrating the relation between the arthroscopic changes of biceps pulley in cases of rotator cuff tear and the concomitant pathology's severity at the anterosuperior region, the arthroscopic changes of pulley were classified into four types, and the author considered that type 1 (normal stretch) and type 2 (minor changes) were lesions with intact pulleys that did not affect biceps stability. The biceps pulley lesions (type 3 and type 4) were detected in 48% (282 of 589 patients) of rotator cuff tear cases. Despite the overall incidence of pulley lesions in their study [11] being less than that in the current study, the incidence of types III and IV was comparable.

Regarding the accuracy of MRI in diagnosing biceps pulley lesions, subluxation and dislocation of the long head of the biceps tendon in relation to the bicipital groove can be detected. In the current study, no specific signs of the pulley lesions were reported in the conventional MRI to diagnose rotator cuff tears in the examined cases, with the limited value of MRI in detecting pulley lesions. This result was comparable to the Baumann et al. [16] retrospective study, which assessed the prevalence of isolated pulley lesions in around a thousand cases subjected to diagnostic shoulder arthroscopy. On the other hand, in a study [17] evaluating the magnetic resonance arthrography (MRA) diagnostic accuracy in biceps pulley lesions, 80 patients were investigated with MRA and subjected to arthroscopy within 2-3 months. The MRA of 28 patients with an injured pulley confirmed by arthroscopy was retrospectively evaluated for pulley lesion presence by three radiologists who did not know the arthroscopic results. The MRA showed a sensitivity of 85.5% and a specificity of 93.5% in the detection of biceps pulley lesions, concluding that the MRA of the shoulder showed high accuracy in biceps pulley lesions assessment.

Our study found that age had a statistical significance in the incidence of pulley lesions, with older patients having a higher incidence. Age was reported to be the most powerful factor in rotator cuff disease and its progression [13,18]. In the current study, the chance of surgical intervention by tenotomy or tenodesis increased in older patients as the incidence of pulley lesions and long head of the biceps tendon lesions was higher. Arthroscopic tenotomy or tenodesis of the biceps tendon was done for all patients with injured pulleys above the age of 50 years, while intervention to the tendon was done in only 65% of patients (15 out of 23) in 40 to 50 years. These were in line with previous research, as all patients above 50 underwent surgical treatment of the biceps tendon [19]. It was previously reported that both procedures were valuable in simultaneous rotator cuff repair in terms of function, pain, and range of motion. Meanwhile, tenotomy had decreased abduction strength [19].

Our study had several strengths, as we had comprehensive data collection by utilizing extensive data collection methods to ensure robust and reliable results. We also included a diverse sample population, which enhanced the generalizability of the findings. Our study also had several limitations: despite the efforts to include a diverse population, the overall sample size was relatively small, which may limit the generalizability of the results. We also had geographical limitations, as our study was conducted in a specific geographical area, which might not reflect the conditions in other regions. Our study also focused on specific aspects of the broader research question, potentially overlooking other influential factors.

## Conclusions

The overall incidence of biceps pulley lesions in cases that underwent arthroscopic cuff repair was remarkably high at 85%. The findings revealed a significant correlation between patient age and the incidence of pulley lesions; older patients with cuff tears were more likely to have these lesions identified during arthroscopic surgery. This suggests that as patients age, the structural integrity of the biceps pulley system may be more susceptible to damage, thereby increasing the likelihood of encountering such lesions during surgical interventions. Consequently, the need for surgical management also rises with age, underscoring the importance of careful assessment and consideration of potential pulley lesions in older patients undergoing cuff repair.

Conversely, the study found no significant associations between the incidence of biceps pulley lesions and factors such as gender or the mode of injury leading to the cuff tear. This indicates that while demographic and injury-related variables might influence other aspects of rotator cuff pathology, they do not appear to impact the occurrence of pulley lesions significantly. Additionally, the limited role of MRI in diagnosing biceps pulley lesions was highlighted, suggesting that more reliance on this imaging modality is needed for accurate detection. These findings emphasize the importance of a thorough arthroscopic examination to identify and appropriately manage biceps pulley lesions, especially in older patients with rotator cuff tears.

## References

[1] Ahmad ZY, Diaz LE, Roemer FW, Goud A, Guermazi A (2022). Imaging review of subscapularis tendon and rotator interval pathology. Radiol Res Pract.

[2] Gohlke F, Essigkrug B, Schmitz F (1994). The pattern of the collagen fiber bundles of the capsule of the glenohumeral joint. Jr Sho Elb Sur.

[3] Morag Y, Jacobson JA, Shields G, Rajani R, Jamadar DA, Miller B, Hayes CW (2005). MR arthrography of rotator interval, long head of the biceps brachii, and biceps pulley of the shoulder. Radiology.

[4] Petchprapa CN, Beltran LS, Jazrawi LM, Kwon YW, Babb JS, Recht MP (2010). The rotator interval: a review of anatomy, function, and normal and abnormal MRI appearance. AJR Am J Roentgenol.

[5] Woertler K (2015). Rotator interval. Semin Musculoskelet Radiol.

[6] Nakata W, Katou S, Fujita A, Nakata M, Lefor AT, Sugimoto H (2011). Biceps pulley: normal anatomy and associated lesions at MR arthrography. Radiographics.

[7] Habermeyer P, Magosch P, Pritsch M, Scheibel MT, Lichtenberg S (2004). Anterosuperior impingement of the shoulder as a result of pulley lesions: a prospective arthroscopic study. J Shoulder Elbow Surg.

[8] Le Huec JC, Schaeverbeke T, Moinard M (1996). Traumatic tear of the rotator interval. J Shoulder Elbow Surg.

[9] Braun S, Horan MP, Elser F, Millett PJ (2011). Lesions of the biceps pulley. Am J Sports Med.

[10] Martetschläger F, Zampeli F, Tauber M, Habermeyer P (2020). Lesions of the biceps pulley: a prospective study and classification update. JSES Int.

[11] Choi CH, Kim SS, Kim SJ, Lee JH (2015). Arthroscopic changes of the biceps pulley in rotator cuff tear and its clinical significance in relation to treatment. Clin Orthop Surg.

[12] Codding JL, Keener JD (2018). Natural history of degenerative rotator cuff tears. Curr Rev Musculoskelet Med.

[13] Abate M, Di Carlo L, Salini V, Schiavone C (2017). Risk factors associated to bilateral rotator cuff tears. Orthop Traumatol Surg Res.

[14] von Elm E, Altman DG, Egger M, Pocock SJ, Gøtzsche PC, Vandenbroucke JP (2008). The strengthening the reporting of observational studies in epidemiology (STROBE) statement: guidelines for reporting observational studies. J Clin Epidemiol.

[15] Hawi N, Liodakis E, Garving C, Habermeyer P, Tauber M (2017). Pulley lesions in rotator cuff tears: prevalence, etiology, and concomitant pathologies. Arch Orthop Trauma Surg.

[16] Baumann B, Genning K, Böhm D, Rolf O, Gohlke F (2008). Arthroscopic prevalence of pulley lesions in 1007 consecutive patients. J Shoulder Elbow Surg.

[17] Schaeffeler C, Waldt S, Holzapfel K (2012). Lesions of the biceps pulley: diagnostic accuracy of MR arthrography of the shoulder and evaluation of previously described and new diagnostic signs. Radiology.

[18] May T, Garmel GM (2024). Rotator Cuff Injury. https://www.ncbi.nlm.nih.gov/books/NBK547664/..

[19] Meraner D, Sternberg C, Vega J, Hahne J, Kleine M, Leuzinger J (2016). Arthroscopic tenodesis versus tenotomy of the long head of biceps tendon in simultaneous rotator cuff repair. Arch Orthop Trauma Surg.

